# Identifying interindividual variability of social perception and associated brain anatomical correlations in children with autism spectrum disorder using eye-tracking and diffusion tensor imaging MRI (DTI-MRI)

**DOI:** 10.1093/cercor/bhad434

**Published:** 2023-11-30

**Authors:** Alice Vinçon-Leite, Ana Saitovitch, Herve Lemaître, Elza Rechtman, Jennifer Boisgontier, Ludovic Fillon, Anne Philippe, Marlène Rio, Isabelle Desguerre, Aurélie Fabre, Khawla Aljabali, Nathalie Boddaert, Monica Zilbovicius

**Affiliations:** Institut Imagine, UMR 1163, INSERM U1299, Department of Pediatric Radiology, Necker-Enfants Malades Hospital, AP-HP, Université Paris Cité, 75015 Paris, France; Department for Autism, SATORI, Henri Guérin Hospital, Pierrefeu du Var 83390, France; Institut Imagine, UMR 1163, INSERM U1299, Department of Pediatric Radiology, Necker-Enfants Malades Hospital, AP-HP, Université Paris Cité, 75015 Paris, France; Institut des Maladies Neurodégénératives, CNRS UMR 5293, Université de bordeaux, Centre Broca Nouvelle-Aquitaine, Bordeaux, France; Department of Environmental Medicine and Public Health, Icahn School of Medecine at Mount Sinai, New York, NY 10029, United States; Institut Imagine, UMR 1163, INSERM U1299, Department of Pediatric Radiology, Necker-Enfants Malades Hospital, AP-HP, Université Paris Cité, 75015 Paris, France; Institut Imagine, UMR 1163, INSERM U1299, Department of Pediatric Radiology, Necker-Enfants Malades Hospital, AP-HP, Université Paris Cité, 75015 Paris, France; Developmental Brain Disorders Laboratory, Imagine Institute, Université Paris Cité, 75015 Paris, France; Service de Médecine Génomique des Maladies Rares, Hôpital Necker-Enfants Malades, APHP-Centre, Paris, France. Laboratoire de génétique des troubles du neurodéveloppement, Institut Imagine, Université de Paris, 75015 Paris, France; Paediatric Neurology Department, Necker-Enfants malades University Hospital, Assistance Publique Hôpitaux de Paris, Paris Cité University, 75015 Paris, France; Institut Imagine, UMR 1163, INSERM U1299, Department of Pediatric Radiology, Necker-Enfants Malades Hospital, AP-HP, Université Paris Cité, 75015 Paris, France; Institut Imagine, UMR 1163, INSERM U1299, Department of Pediatric Radiology, Necker-Enfants Malades Hospital, AP-HP, Université Paris Cité, 75015 Paris, France; Institut Imagine, UMR 1163, INSERM U1299, Department of Pediatric Radiology, Necker-Enfants Malades Hospital, AP-HP, Université Paris Cité, 75015 Paris, France; Institut Imagine, UMR 1163, INSERM U1299, Department of Pediatric Radiology, Necker-Enfants Malades Hospital, AP-HP, Université Paris Cité, 75015 Paris, France

**Keywords:** autism spectrum disorder, eye-tracking, DTI-MRI, fractional anisotropy, social perception

## Abstract

Even though deficits in social cognition constitute a core characteristic of autism spectrum disorders, a large heterogeneity exists regarding individual social performances and its neural basis remains poorly investigated. Here, we used eye-tracking to objectively measure interindividual variability in social perception and its correlation with white matter microstructure, measured with diffusion tensor imaging MRI, in 25 children with autism spectrum disorder (8.5 ± 3.8 years). Beyond confirming deficits in social perception in participants with autism spectrum disorder compared 24 typically developing controls (10.5 ± 2.9 years), results revealed a large interindividual variability of such behavior among individuals with autism spectrum disorder. Whole-brain analysis showed in both autism spectrum disorder and typically developing groups a positive correlation between number of fixations to the eyes and fractional anisotropy values mainly in right and left superior longitudinal tracts. In children with autism spectrum disorder a correlation was also observed in right and left inferior longitudinal tracts. Importantly, a significant interaction between group and number of fixations to the eyes was observed within the anterior portion of the right inferior longitudinal fasciculus, mainly in the right anterior temporal region. This additional correlation in a supplementary region suggests the existence of a compensatory brain mechanism, which may support enhanced performance in social perception among children with autism spectrum disorder.

## Introduction

Autism spectrum disorder (ASD) is a neurodevelopmental disorder that is characterized by social communication and interaction difficulties, as well as restricted and repetitive patterns of behavior, interests, or activities (American Psychiatric Association 2013). In spite of a large heterogeneity in clinical manifestations, deficits in social interactions constitute a core characteristic of ASD. For several years now, social perception abnormalities in ASD have been objectively characterized with eye-tracking technology. Indeed, a large number of eye-tracking studies confirmed an atypical gaze pattern when exploring social stimuli in children, adolescents, and adults with ASD (Klin et al. 2002; Shic et al. 2011; Hosozawa et al. 2012; Rice et al. 2012; Chawarska et al. 2013; Frost-Karlsson et al. 2019; Tang et al. 2019; Kaliukhovich et al. 2021). A meta-analysis of 38 published eye-tracking studies confirmed that subjects with ASD attend definitively less to social stimuli compared with typically developing (TD) individuals (Chita-Tegmark 2016).

More recently, studies have indicated that social perception measures obtained using eye-tracking could be a potential biomarker for ASD (Murias et al. 2018). In addition, by exploring a large sample of nearly 2,000 toddlers using a categorical approach, Wen and colleagues showed that it was possible to identify subgroups based on eye-tracking data (Wen et al. 2022). Still, there are no eye-tracking studies today using a dimensional approach to investigate the distribution of interindividual differences in social perception within the autism spectrum. Indeed, description of a putative continuous distribution of social perception deficits could help to further characterize social impairments in autism.

Moreover, this dimensional characterization of interindividual differences in social perception could provide valuable information contributing to further characterization of the neural basis of the social impairments in ASD. Indeed, combining eye-tracking with neuroimaging investigation could provide a more comprehensive understanding of the neural mechanisms underlying social perception. This approach can help to identify brain regions that are involved in social perception and how they may differ across individuals. For the last 30 years, results from a large number of studies have consistently described anatomo-functional abnormalities within the social brain network in children and adults with ASD compared with TD controls (Saitovitch et al. 2012; Sato and Uono 2019). However, till now, no study has explored the relationship between eye-tracking and neuroimaging data in ASD.

In line with this approach, we have recently used eye-tracking to investigate interindividual differences in social perception in children and adolescents with typical development. Results showed that there is a continuous variation in social perception across individuals. Indeed, a large interindividual variability, following a Gaussian distribution, was observed in the number of fixations to the eyes of characters during visualization of social scenes. In addition, we have performed a study combining the eye-tracking data and measures of fractional anisotropy (FA), an index of white matter (WM) microstructure integrity obtained with diffusion tensor imaging (DTI-MRI). Whole brain analysis showed a significant positive correlation between FA and number of fixations to the eyes of characters, mainly in the temporal part of the superior longitudinal fasciculi bilaterally, adjacent to the posterior superior temporal cortex. Children and adolescents who looked more to the eyes of characters were those who had higher FA values within these tracts (Vinçon-Leite et al. 2020). These results contribute to a better understanding of the neural basis of social perception in typical development and highlight the relevance of expanding this approach to ASD.

In this context, in the present study, we specifically tested three hypotheses: (i) it is possible to use eye-tracking to objectively describe a putative continuous distribution in interindividual variability in social perception in children and adolescents with ASD, as previously reported in TD children and adolescents; (ii) this interindividual variability in social perception would be correlated with WM microstructure, measured with DTI-MRI, particularly in tracts connecting cortical areas implicated in social cognition processes, namely the social brain network; (iii) finally, considering social perception and WM abnormalities previously described in ASD, we expect to find a different correlation pattern between eye-tracking and DTI-MRI data in children and adolescents with ASD compared with TD controls.

## Materials and methods

### Participants

Twenty-five children and adolescents with a clinical diagnosis of ASD (18 males, 7 females; age = 8.5 ± 3.8 years; range: 2.4–16 years) participated in this study and were recruited in university children’s hospitals, designated as reference centers for autism diagnosis by the French Health Ministry. The diagnosis of ASD was stablished by a multidisciplinary team, including senior child psychiatrists and child psychologists. The clinician judgment was informed by the DSM-IV-revised and DSM-5 criteria (diagnosis statistical manual; American Psychiatric Association 2013) as well as by the Autism Diagnostic Interview-Revised (ADI-R; Lord et al. 1994). Exclusion criteria were any medical and/or genetic condition accounting for the autistic symptoms, as well as prematurity and prenatal or perinatal problems.

Twenty-four TD children and adolescents (15 males, 9 females, age = 10.5 ± years, range: 6.0–17.4 years) participated in this study and were recruited using an advertisement. It consists on the same sample as in our previous study (Vinçon-Leite et al. 2020). All TD participants were free of psychiatric, neurological, and general health problems, as well as any learning disabilities. No participant presented a medical history of prematurity or any kind of prenatal or perinatal problems. All of them had a normal scholarship.

Demographic data are summarized in Table 1.

**Table 1:** Demographic data.

	**TD (*n* = 24)**	**ASD (*n* = 25)**	***P*-value**
Age in years			0.04 (*t*-test)
Mean ± SD	10.5 ± 2.9	8.5 ± 3.8	
Min – Max	6.0–17.4	2.4–16	
Sex			0.7 (chi-square test)
Female	9 (37.5%)	7 (28%)	
Male	15 (62.5%)	18 (72%)	
IQ			0.002 (*t*-test)
Mean ± SD	112.2 ± 12.0	90.5 ± 29.1	
ADI-R score	_____	41.9 ± 10.4	

All participants (ASD and TD) presented no contraindication for the MRI scan and had normal or corrected-to-normal vision. Written informed consent to participate to this study was provided by all participant parents or legal guardian and adhered to the principles of Helsinki Declarations. The study was approved by the Research Ethical Committee of Necker Hospital, Paris, France. All experiments were performed in accordance with the ethical guidelines and regulations.

### Eye-tracking protocol

#### Experimental design and acquisition

The study was performed using the Tobii™ T120 eye-tracker equipment, based on infra-red technology, consisting of a 17-inch TFT monitor with a resolution of 1280 × 1024 pixels, from which the stimuli were presented in full screen, and the gaze behavior was simultaneously recorded. The eye-tracking system was completely noninvasive with little indication that the eye-movements were being tracked and with no artificial constraints of the head or body movements. The system tracked both eyes to a rated accuracy of 0.5° with a sampling rate of 60 Hz. The Tobii™ equipment was connected to an HP Pavillon DV6 laptop computer (Windows 7 Professional).

The participants were individually tested, seated facing the eye-tracker monitor at approximatively 60 cm; the experimenter sat next to the participant to control the computer without interfering with the viewing behavior. A calibration test consisting of five registration points was performed before each set of stimuli. The calibration test was repeated if the examiner considered one of the five points not valid according to the eye-tracker criteria. The participants were instructed that they would see a sequence of movie fragments and all they had to do was to watch them. The stimuli creation, the calibration procedures and the data acquisition and visualization were performed using Tobii Studio™ software.

#### Stimuli

To investigate the individual variability in eye-gaze behavior, we used an eye-tracking paradigm developed in our lab (Saitovitch et al. 2016; Saitovitch et al. 2019). The eye-tracking task was a passive visualization of naturalistic social movies; no specific task performance was required. To provide an ecological and naturalistic setting, we used 5 short movie fragments (10 s) extracted from the commercial movie (Le Petit Nicolas) displaying social scenes with characters engaged in peer-to-peer social interactions. Sounds in the movies were dialogs and factors as scene background and characters’ position were not controlled for.

#### Eye-tracking recording validity

For eye-tracking recordings, a threshold of 50% of valid data was established. All participants included in this study matched such recording quality criteria, based on the amount of valid and missing data, indicated by Tobii studio software. In addition, all recordings were visually checked by two clinicians (Dr M.Z and Dr A.V.L) including an expert in eye-tracking acquisition (Dr M.Z). After these quantitative and visual quality control on eye-tracking data, 4/29 participants with ASD were excluded because of excessive motion during the eye-tracking recording leading to poor data quality.

### DTI-MRI protocol

#### MRI acquisition

Separately from the eye-tracking session, all participants underwent a cerebral MRI scan in which DTI was used to measure FA, as an index of brain WM microstructural integrity. This study was monocentric. All brain images were acquired within the same GE-Signa 1.5 Tesla MR scanner located at Necker Hospital, in Paris, France, using a 12-channel head coil. Clinical sequences were acquired (3DT1, coronal T2, and coronal FLAIR) to ensure participants presented no radiological brain abnormalities. Diffusion-weighted images were acquired using an echo-planar imaging sequence (axial slices, echo time (TE) ≈ 96 milliseconds, repetition time (TR) = 15,000 ms, 40 diffusion encoding directions with b-value = 1,000 s/mm^2^ and one *b*-value = 0 s/mm^2^, voxel size: 2.4 × 2 × 2 mm^3^), adapted to tensor measurements.

#### Diffusion imaging preprocessing

First, all raw diffusion images were visually checked for major artifacts. Then, diffusion data were preprocessed using the DESIGNER pipeline (Ades-Aron et al. 2018) with tools available in MRtrix3 package (Tournier et al. 2019) in order to denoise, dering, and bias correct the raw images. Indeed, to reduce the noise effect on the diffusion parameter estimation, the MRtrix3 *dwidenoise* tool (Copyright © 2016 New York University, University of Antwerp, https://github.com/MRtrix3/mrtrix3) was applied as the first step of the preprocessing (Veraart et al. 2016a, 2016b). Then, the Gibbs ringing correction framework of Kellner et al. (Kellner et al. 2016) was applied to remove Gibbs ringing artifacts with *mrdegibbs* tool. Finally, we applied B1 field inhomogeneity correction (Smith et al. 2004) using the *dwibiascorrect* tool. After denoise, degibbs, and bias correct, images were corrected for head motion and eddy-currents using FMRIB Diffusion Toolbox in FMRIB Software Library (FSL) (www.fmrib.ox.ac.uk/fsl). It consisted of an affine registration to the first *b* = 0 image for head motion and eddy currents corrections. Gradient tables were reoriented accordingly to the affine transformation. We then performed a brain extraction using the Brain Extraction Tool, and a voxel-wise diffusion tensor fitting to obtain images of FA. Furthermore, for quality improvement, we performed Robust Estimation of Tensors by Outlier Rejection on a voxel-wise basis to identify and exclude outliers from the multiple diffusion directions collected, and to calculate the diffusion tensor from the remaining data to provide more robust estimates of diffusion parameters than other fitting procedures (Chang et al. 2005).

#### Tract-based spatial statistics

Voxel-wise statistical analysis of the FA data was carried out using Tract-based spatial statistics (TBSS; Smith et al. 2006), part of FSL. Firstly, every FA image was aligned to every other one, identifying the “most representative” one and using it as a target image for each group’s correlation analysis. For between groups comparison analysis, we used our population specific template. These target images were then affine-aligned into MNI152 standard space, and every image was transformed into 1 × 1 × 1 mm^3^ MNI152 space by combining the nonlinear transform to the target FA image with the affine transform from that target to MNI152 space. Next, the mean FA image was created and thinned to create a mean FA skeleton, which represents the center of all tracts since center of WM bundles are supposed to present less interindividual variability. The skeleton was then threshold to FA > 0.2 to keep only the main tracts. Each child’s and adolescent’s aligned FA maximum values were then projected onto the skeleton and the resulting data fed into voxel-wise cross-individual statistics. There was no significant difference for whole brain mean FA values between the groups.

#### Data quality control of DTI images

Because head motion can significantly impact DTI results, we performed a strict data quality control on motion parameters (Baum et al. 2018). First, participants were excluded based on visual inspection of diffusion scans for clinical abnormalities and for motion related artifacts or poor scan quality by an expert radiologist (Pr N.B). An automatic quality control was performed during the preprocessing to detect large head motion and signal dropout. After this step, one TD participant (1/25) and one participant with ASD (1/26) were discarded because of excessive head motion and blink-eye artifacts. After running motion and eddy correction using FSL’s *eddy_correct* tool, we extracted how much the data had to be moved to counter head motion. We then used Tromp’s code to calculate the Euclidian distance (Tromp 2016). It consists of taking the absolute value of the *x*, *y*, and *z* translation and calculates the average for each, to calculate total euclidian distance per subject. No significant difference in euclidian distance was found between the two groups (mean = 0.55 ± 0.29; *t*_(40)_ = −1.3; *P* = 0.2). A visual quality control was performed on the FA images for proper spatial normalization and brain masking. The final sample for TBSS analysis was constituted of 25 participants with ASD and 24 TD participants.

### Statistical analysis

#### Eye-tracking data

Gaze patterns were analyzed with dynamic area of interest (AOI) allowing dynamic “frame by frame” measurements throughout the videos. For each movie fragment, the dynamic AOI corresponding to the eye regions of characters was selected for analysis, as a measure of precise social cues perception. Our variable of interest was the number of fixations to the eyes. Eye-tracking software interpolates the shape and position of the AOI, so that it moves smoothly from one frame to the next. Importantly, AOI size and shape remained stable across measurements. The number of fixations in the AOI “eyes” was recorded using the Tobii Studio™ software. A fixation event was defined as such by the Tobii fixation filter based on 0.42 pixels/ms threshold. Absolute number of fixations was selected since it is an absolute variable that varies dimensionally across participants and informs on exploratory behavior toward a defined region: a higher number of fixations indicates that people further explore the region (Davis et al. 2017). The total number of fixations to the eyes for each participant was extracted from Tobii Studio™ and then exported and computed with R cran software (http://www.R-project.org/—version 1.2.1335).

To study interindividual variability in social perception, we measured in each group the distribution of number of fixations to the eyes during visualization of the social movies. Normality of the data was tested using the Shapiro test and Pearson measure of kurtosis was derived from the distribution of the data. In addition, we used a linear regression model to study the number of fixations to the eyes as a function of diagnosis (ASD or TD), adding age and sex as covariates. Statistical threshold was set at *P* < 0.05.

#### Correlation between DTI and eye-tracking data

Using the same strategy applied in our previous study, voxel-wise correlation between number of fixations to the eyes and FA values was performed. Analyses were performed using the number of fixations to the eyes as an explanatory variable to FA values (variable of interest) variation across subjects, in each group (ASD and TD) separately. In addition, the interaction “group by number of fixations to the eyes” was tested. For multiple comparison correction, a statistical threshold was set at *P* < 0.05 with threshold-free cluster enhancement (TFCE) option (Smith and Nichols 2009) and after 10,000 nonparametric permutations using the randomize tool in FSL (Winkler et al. 2014).

Results were presented using Montreal Neurologic Institute (MNI) coordinates. Only clusters with an extent > 160 continuous voxels were considered to eliminate isolated small clusters from further consideration. In addition, we applied a binary mask on the results to exclude the cerebellum that was not uniformly cropped in all participant’s data. The most probable anatomic localization of each significant cluster was determined using The John Hopkins University White Matter tractography atlas tool, in FSL. Finally, the mean FA from the clusters showing significant correlations with number of fixations to the eyes were extracted and then exported to R cran software. Same linear regressions between the number of fixations in the eyes and extracted mean FA values in the significant clusters were also performed adding age and sex as covariate.

## Results

### Eye-tracking results

**Fig. 1:**
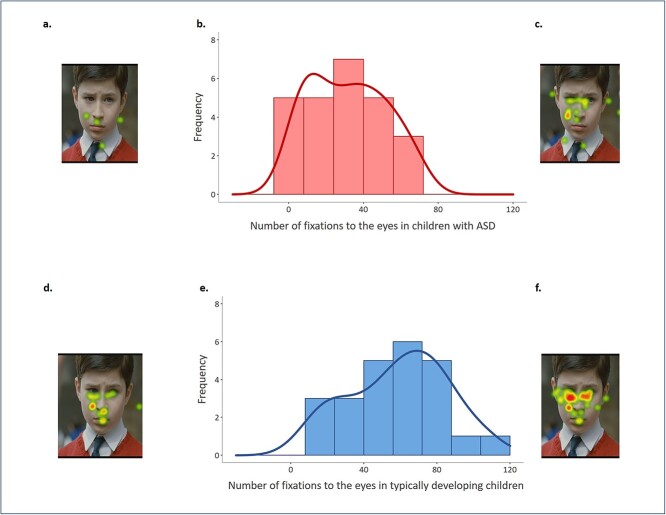
Distribution of interindividual variability in eye-tracking data. Upper panel: children with ASD (*n* = 25). (a) and (c) examples of heat map on number of fixations to the eyes: Warm colors denote more fixations and cold colors denote fewer fixations to the eyes. Scenes were selected for illustrative purpose. (b) The histogram shows the frequency of the number of fixations to the eyes across the group. Lower panel: TD children (*n* = 24). (d and f) Examples of heat map on number of fixations to the eyes: warm colors denote more fixations and cold colors denote fewer fixations to the eyes. Scenes were selected for illustrative purpose. (e) The histogram shows the frequency of number of fixations to the eyes across the group.

In children and adolescents with ASD, we observed that the number of fixations largely differs among participants (mean = 32 ± 20; range: 4–67 fixations), with great interindividual variability in gaze pattern to the eyes during visualization of social movies (kurtosis = −1.2). In addition, the number of fixations to the eyes followed a normal distribution (Shapiro-test *W* = 0.94, *P* = 0.12) (Fig. 1a,b,c). As previously reported, we also observed great interindividual variability in gaze pattern to the eyes following a normal distribution in children and adolescents with typical development (range: 14–106 fixations; kurtosis = −0.88; Shapiro-test *W* = 0.96, *P* = 0.42) (Vinçon-Leite et al. 2020) (Fig. 1d,e,f).

Participants with ASD present a significant decrease in number of fixations to the eyes during visualization of social movies compared with participants with typical development (ASD = 32 ± 20; TD = 59 ± 27; *b* = −22.8, *t*_(45)_ = −3.3, *P* = 0.002, regression). Finally, there was no correlation between number of fixations to the eyes and age of participants in both groups (TD: *F*_(1,22)_ = 1.9, *P* = 0.18; ASD: *F*_(1,23)_ = 3.9, *P* = 0.06; ANOVA).

### Correlation between DTI and eye-tracking data

**Fig. 2:**
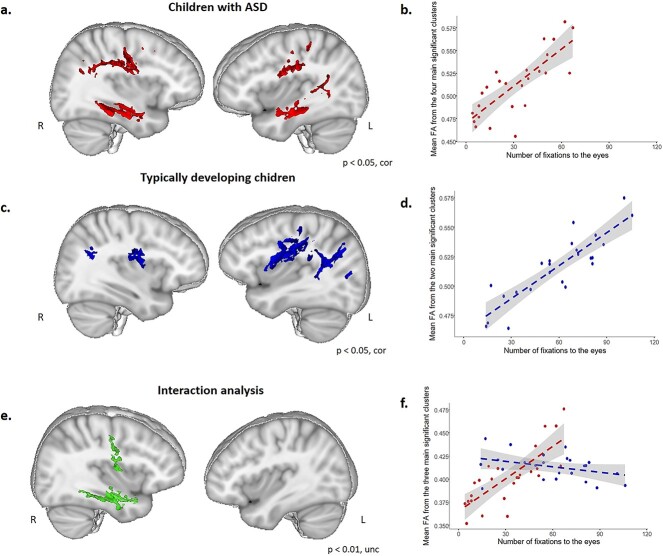
Correlation between the number of fixations to the eyes and FA values in TD children, in children with ASD and interaction analysis (group by number of fixations to the eyes). (a and b) Participants with ASD*.* (a) Significant positive correlation between the number of fixations to the eyes and FA values within the WM skeleton (*P* < 0.05 family wise error and threshold free cluster enhancement corrected for multiple comparisons). Significant correlation was observed in WM tracts mainly encompassing right and left superior longitudinal fasciculus and right and left inferior longitudinal fasciculus. Results were overlaid on the MNI-152 template average brain using mango software including the four significant clusters: Cluster 1 = 11,024 voxels; cluster 2 = 2,108 voxels; cluster 3 = 1,006 voxels; cluster 4 = 854 voxels. Right and left sagittal views (*x* = 35 and −38, respectively). (b) Scatterplot of positive correlation between the average FA from these clusters (TBSS analysis) and the number of fixations to the eyes (*b* = 9.9e-04, *t*(21) = 7.3, *P* = 3.4e-07, regression performed in R with age and sex as covariates). (c and d) Participants with typical development. (c) Significant positive correlation between the number of fixations to the eyes and FA values within the WM skeleton (*P* < 0.05 family wise error and threshold free cluster enhancement corrected for multiple comparisons). Significant correlation was observed in WM tracts mainly encompassing right and left superior longitudinal fasciculus. Results were overlaid on the MNI-152 template average brain using mango software including the two significant clusters: Cluster 1 = 15,399 voxels; cluster 2 = 186 voxels. Right and left sagittal views (*x* = 35 and −38, respectively). (d) Scatterplot of positive correlation between the average FA from these clusters (TBSS analysis) and the number of fixations to the eyes (*b* = 9.0e-04, *t*_(20)_ = 8.4, *P* = 5.77e-08 regression performed in R with age and sex as covariates). (e and f) Interaction analysis. (e) Significant group by number of fixations to the eyes interaction within the WM skeleton (*P* < 0.01 family wise error and threshold free cluster enhancement uncorrected for multiple comparisons), localized in WM tracts mainly encompassing the anterior temporal portion of the right inferior longitudinal fasciculus. Results were overlaid on the MNI-152 template average brain using mango software including the three significant clusters: (cluster 1 = 1,198 voxels; cluster 2 = 431 voxels; cluster 3 = 286 voxels). Right and left sagittal views (*x* = 35 and −38, respectively). (f) Scatterplot of significant group by number of fixations to the eyes interaction from these clusters (TBSS analysis) (*F*_(1,43)_ = 50.9; *P* = 8.24e-09, regression adding age and sex as covariates) performed in R.

In participants with ASD, whole brain voxel-by-voxel analyses showed significant positive correlation between the number of fixations to the eyes and FA in extended WM tracts (*P* < 0.05, TFCE-corrected). This significant correlation was found within four main clusters (cluster 1: 11,024 voxels, location of the maximum intensity voxel at *x* = 5, *y* = −14, *z* = 24, *P* = 0.011; cluster 2: 2,821 voxels, location of the maximum intensity voxel at *x* = 6, *y* = −17, *z* = −2, *P* = 0.032; cluster 3: 1,006 voxels, location of the maximum intensity voxel at *x* = 44, *y* = −34, *z* = −12, *P* = 0.031; cluster 4: 854 voxels, location of the maximum intensity voxel at *x* = −42, *y* = −15, *z* = −19, *P* = 0.033, Fig. 2a) Anatomically distinct WM pathways were identified in these clusters mainly encompassing: right and left superior longitudinal fasciculus bilaterally nearby the posterior part of the arcuate fasciculi, right and left inferior longitudinal fasciculus and left IFOF (inferior fronto-occipital fasciculus) and right and left anterior thalamic radiation and corticospinal tracts (Fig. 2a, see Supplementary Data Tables S1 and S4). Participants with ASD who looked more to the eyes of characters during visualization of social movies are those with higher FA values in WM tracts encompassing these tracts (*b* = 9.9e-04, *t*_(21)_ = 7.3, *P* = 3.4e-07) (Fig. 2b).

In participants with typical development, as previously described (Vinçon-Leite et al. 2020), whole brain voxel-by-voxel analyses showed a significant positive correlation between the number of fixations to the eyes and FA in localized WM tracts (*P* < 0.05, TFCE-corrected). This significant correlation was found within two main clusters (cluster 1: 15,399 voxels, location of the maximum intensity voxel at *x* = − 35, *y* = − 50, *z* = 31, *P* = 0.017; cluster 2: 186 voxels, maximum intensity voxel at *x* = 36, *y* = − 47, *z* = 26, *P* = 0.045) (Fig. 2c). Anatomically distinct WM pathways were identifiable in these two principal clusters mainly encompassing: the superior longitudinal fasciculus and its temporal part localized nearby cortical temporo-parietal junction bilaterally, the corpus callosum (forceps major), and right and left anterior thalamic radiations and the left corticospinal tract (Fig. 2c, see Supplementary Material Tables S2 and S5). TD participants who looked more to the eyes of characters during visualization of social movies are those who had significantly higher FA values in these circumscribed WM tracts (*b* = 9.0e-04, *t*_(20)_ = 8.4, *P* = 5.77e-08, regression) (Fig. 2d). We did not find any significant negative correlation between FA values and the number of fixations to the eyes.

Whole brain voxel-by-voxel analyses showed a significant interaction between group and number of fixations to the eyes (*P* < 0.01, TFCE, unc), localized in three main clusters (cluster 1: 1,198 voxels, maximum intensity voxel at *x* = 22, *y* = −13, *z* = −26, *P* = 0.007; cluster 2, 431 voxels, maximum intensity voxel at *x* = 48, *y* = 3, *z* = 10, *P* = 0.008, cluster 3; 286 voxels, maximum intensity voxel at *x* = 37, *y* = 14, *z* = 40, *P* = 0.008) (Fig. 2e). Anatomically distinct WM pathways were identifiable in these clusters mainly encompassing the anterior temporal portion of the right inferior longitudinal fasciculus (Fig. 2e, see Supplementary Data Tables S3 and S6). Participants with ASD who looked more to the eyes of characters during passive visualization of social movies are those with higher FA values within these WM clusters whereas in TD participants FA values remain stable independently of number of fixations to the eyes (*F*_(1,43)_ = 50.9 *P* = 8.24e-09) (Fig. 2f).

Additional results regarding radial diffusivity and mean diffusivity are presented as supplementary material.

## Discussion

To the best of our knowledge, this is the first study that investigated interindividual variability in gaze behavior in children and adolescents with ASD, and its association with brain WM microstructure integrity. Firstly, confirming our hypothesis, the results obtained with eye-tracking allowed to describe a continuous distribution in interindividual variability in social perception in children and adolescents with ASD. In addition, whole brain analysis showed a significant positive correlation between FA and number of fixations to the eyes of characters mainly in tracts connecting cortical area implicated in social cognition processes, namely the social brain network. Importantly, better performance in social perception in children with ASD was associated with higher FA values in a supplementary region compared with TD controls, mainly localized in the right temporal pole.

In this study, a classical group analysis comparing eye-tracking data from children with ASD and TD children showed a lack of preference for the eyes in ASD. Furthermore, exploring the eye-tracking data using a dimensional approach allowed to describe a large interindividual variability in social perception in children with ASD. During visualization of social scenes, some children with ASD look more to the eyes of characters while others look less. Importantly, this interindividual variability followed a normal distribution indicating a spectrum of social perception deficits, which is in agreement with the concept of “spectrum” in ASD. Within this same perspective, we have also described interindividual variability in social perception in typical development. Our results showed that, when viewing social scenes, some individuals look more to the eyes of characters while others look less, all remaining within a spectrum of typical social functioning. Even though the distribution of eye-tracking data followed a similar pattern in both groups, the horizontal displacement of the curbs illustrates the deficits that can be observed in ASD (Fig. 1).

Abnormalities in gaze behavior toward socially relevant information in ASD have been extensively investigated over the last 20 years using eye-tracking techniques. A large number of studies have showed an atypical gaze pattern, characterized by a lack of preference for socially relevant information, in children and adults with ASD (Papagiannopoulou et al. 2014; Chita-Tegmark 2016, for review). More recently, eye-tracking has emerged as a potential biomarker for autism. Studies have shown that abnormal gaze pattern toward socially relevant stimuli in early childhood could predict later ASD diagnosis (Bacon et al. 2020). Recent results have also shown that eye-tracking data could help to better characterize the heterogeneity in ASD, allowing to identify subgroups, in a categorical approach (Wen et al. 2022). Within the same perspective, using a dimensional approach, our results provide complementary information by describing a spectrum of social perception deficits.

Importantly, our eye-tracking results have provided suitable data allowing to further investigate the neural basis of social perception deficits in ASD. Indeed, the present study is, to our knowledge, the first study to perform a direct correlation between eye-tracking data and measures of FA, an index of WM microstructure, obtained with DTI-MRI. Whole brain analysis showed a significant positive correlation between FA values and the number of fixations to the eyes in WM tracts mainly encompassing right and left superior longitudinal tracts and right and left inferior longitudinal tracts (Fig. 2a). Children with ASD who look more to the eyes of characters are those presenting higher FA values in these tracts, that encompass temporo-parietal junction, connecting temporal and frontal regions, which are key regions for social cognition. The same analysis performed in TD children had shown significant correlation between FA values and the number of fixations to the eyes also in the superior longitudinal fasciculus, but not in inferior longitudinal tracts (Fig. 2b). These results indicate that, similar to children with typical development, higher number of fixations to the eyes in children with ASD is associated with higher FA values in superior temporal tracts. However, and more importantly, in children with ASD, better performance in social perception was also associated with higher FA values in a supplementary region, mainly localized in the right temporal pole. Indeed, results from interaction analysis highlighted that there is a significant difference localized in this region, where a correlation between number of fixations to the eyes and FA exist in children with ASD but not in TD children.

Taken together, the present results could suggest that a compensatory mechanism would take place during the developmental trajectory of children with ASD. This is strongly supported by the fact that WM changes are experience-dependent and have been well stablished as a critical contributing aspect of neuroplasticity in the brain (Sampaio-Baptista and Johansen-Berg 2017). Interestingly, in line with our results, using a functional connectivity approach Jasmin and colleagues have also recently described a neural compensatory strategy in ASD regarding social communication: objective measures of better performance in social communication were associated with higher functional connectivity in specific social brain networks in participants with ASD (Jasmin et al. 2023).

The study of neuroplasticity focusing on WM has only recently started to be invested and can bring valuable information regarding the anatomical architecture and connections supporting clinical and behavioral changes (Boltzmann et al. 2017). The development of tract-specific WM pathways associated with clinical and behavioral improvements has also been described in other contexts. For instance, an enhancement in WM micro-structure was described associated with reading learning in children and adults (Boltzmann et al. 2017; Ekerdt et al. 2020). Recent studies have also shown improved WM both in motor- and non-motor-related structures in association with improved clinical scores in patients with Parkinson disease (Sanjari Moghaddam et al. 2020). The results presented here are in line with this approach, presenting data supporting WM neural plasticity associated with interindividual differences in social performances in ASD. Going beyond description of brain abnormalities, this supports an alternative research path on neuroimaging investigations in ASD, focusing on the description of neural mechanisms associated with better performance or clinical improvement.

One limitation of the present study concerns the sample size. The findings described here were obtained in a limited number of subjects; therefore, they need to be replicated in a large sample. As anticipated within our samples’ specific age range, age has an effect on FA values, that aligns with the established findings in the existing literature (Lebel et al. 2019), but the age difference in our sample has been mostly accounted for by including age in our statistical models. Limitations linked to the DTI methods should also been considered. Indeed, although the traditional tensor model offers readily accessible tensor-derived measures, employing sequences with greater encoding directions and b-values could effectively tackle the issue of crossing fibers and facilitate exploration of more advanced diffusion measures and should be considered in future studies. Further studies could also beneficiate from using age specific tractography atlas, that are currently under investigation in the literature (Short et al. 2022; Spencer et al. 2022) to enhance the neuroanatomical correspondence of WM tracts for this age range. Finally, future longitudinal studies will provide crucial information for understanding the neural processes that support improvements in social deficits in ASD.

Overall, our results suggest that better performance in social cognition in children with ASD, acquired during development, would be associated with the recruitment of an additional region that supports this enhanced performance. This could indicate that, in ASD, neural connections may reorganize and adapt to overcome deficits in social cognition. It is important to note that compensatory mechanism may vary across individual developmental trajectories. Therefore, further research, mainly using a longitudinal approach, could be valuable to help identify the factors that contribute to compensatory mechanisms and their potential implications for treatment and intervention strategies.

## Supplementary Material

supplementary_material-final_version_bhad434

## Data Availability

The data that support the findings of this study are available upon request.

## CRediT statement

Alice Vinçon-Leite (Data curation, Formal analysis, Funding acquisition, Writing—original draft), Ana Saitovitch (Conceptualization, Data curation, Formal analysis, Funding acquisition, Investigation, Methodology, Supervision, Validation, Visualization, Writing—original draft, Writing—review and editing), Herve Lemaitre (Formal analysis, Investigation, Methodology, Software, Supervision, Validation, Visualization), Elza Rechtman (Conceptualization, Data curation, Investigation), Jennifer Boisgontier (Formal analysis, Methodology), Ludovic Fillon (Formal analysis, Software), Anne Philippe (Data curation), Marlène Rio (Data curation), Isabelles Desguerre (Data curation), Aurélie Fabre (Visualization), Khawla Aljabali (Visualization), Nathalie Boddaert (Conceptualization, Data curation, Supervision, Validation, Writing—review and editing), Monica Zilbovicius (Conceptualization, Data curation, Formal analysis, Funding acquisition, Investigation, Methodology, Supervision, Validation, Writing—original draft, Writing—review and editing).
