# Supplementary material for: Identifying interindividual variability of social perception and associated brain anatomical correlations in children with autism spectrum disorder using eye-tracking and diffusion tensor imaging MRI (DTI-MRI)
Source: Cereb Cortex. 2023 Nov 30;34(1):bhad434. doi: 10.1093/cercor/bhad434 (PMC10793563; doi:10.1093/cercor/bhad434)
Supplement: supplementary_material-final_version_bhad434 [file supplementary_material-final_version_bhad434.docx]

**Supplementary Material**

| ASD | White-matter tracts  JHU white matter tractography atlas | Mean probabilities^a^ |
| --- | --- | --- |
| Cluster 1 | Superior longitudinal fasciculus R | 4,0 |
|  | Superior longitudinal fasciculus L | 3,1 |
|  | Corticospinal tract L | 1,7 |
|  | Superior longitudinal fasciculus (temporal part) R | 1,3 |
|  | Superior longitudinal fasciculus (temporal part) L | 1,3 |
|  | Forceps minor | 1,1 |
|  | Corticospinal tract R | 0,9 |
|  | Inferior longitudinal fasciculus L | 0,3 |
|  | Inferior fronto-occipital fasciculus L | 0,3 |
|  | Cingulum (cingulate gyrus) L | 0,2 |
|  | Anterior thalamic radiation L | 0,2 |
|  | Inferior fronto-occipital fasciculus R | 0,1 |
|  |  |  |
| Cluster 2 | Anterior thalamic radiation R | 13,7 |
|  | Anterior thalamic radiation L | 6,4 |
|  | Corticospinal tract R | 3,1 |
|  | Inferior fronto-occipital fasciculus R | 0,1 |
|  |  |  |
| Cluster 3 | Inferior longitudinal fasciculus R | 15,9 |
|  | Inferior fronto-occipital fasciculus R | 3,3 |
|  | Superior longitudinal fasciculus (temporal part) R | 1,5 |
|  | Superior longitudinal fasciculus R | 1,3 |
|  | Uncinate fasciculus R | 0,8 |
|  | Cingulum (hippocampus) R | 0,1 |
|  |  |  |
| Cluster 4 | Inferior longitudinal fasciculus L | 20,7 |
|  | Inferior fronto-occipital fasciculus L | 11,5 |
|  | Uncinate fasciculus L | 1,7 |
|  | Superior longitudinal fasciculus (temporal part) L | 0,9 |
|  | Superior longitudinal fasciculus L | 0,9 |
|  | Anterior thalamic radiation L | 0,6 |

**Supplementary Table S1: Correlation between FA and number of fixations to the eyes (N= 25 participants with ASD)**

^a^ Mean probabilities of the considered tract within the mask of results (outputs from ‘autoaq’ of FSL) in order of importance

**Supplementary Table S2: Correlation between FA and number of fixations to the eyes (N= 24 healthy participants)**

^a^ Mean probabilities of the considered tract within the mask of results (outputs from ‘autoaq’ of FSL) in order of importance

| TD | White-matter tracts  JHU white matter tractography atlas | Mean probabilities^a^ |
| --- | --- | --- |
| Cluster 1 | Superior longitudinal fasciculus L | 4,9 |
|  | Superior longitudinal fasciculus (temporal part) L | 2,0 |
|  | Anterior thalamic radiation L | 1,9 |
|  | Anterior thalamic radiation R | 1,9 |
|  | Corticospinal tract L | 1,6 |
|  | Forceps major | 1,1 |
|  | Forceps minor | 0,8 |
|  | Cingulum (cingulate gyrus) L | 0,7 |
|  | Inferior fronto-occipital fasciculus L | 0,5 |
|  | Superior longitudinal fasciculus R | 0,5 |
|  | Inferior longitudinal fasciculus L | 0,4 |
|  | Inferior fronto-occipital fasciculus R | 0,2 |
|  | Corticospinal tract R | 0,2 |
|  | Superior longitudinal fasciculus (temporal part) R | 0,1 |
|  | Uncinate fasciculus L | 0,1 |
|  |  |  |
| Cluster 2 | Superior longitudinal fasciculus R | 15,8 |
|  | Superior longitudinal fasciculus (temporal part) R | 2,4 |
|  | Inferior longitudinal fasciculus R | 0,2 |

**Supplementary Table S3: Interaction group by number of fixations to the eyes (N= 25 participants with ASD and 24 healthy participants )**

^a^ Mean probabilities of the considered tract within the mask of results (outputs from ‘autoaq’ of FSL) in order of importance

| Interaction | White-matter tracts  JHU white matter tractography atlas | Mean probabilities^a^ |
| --- | --- | --- |
| Cluster 1 | Inferior longitudinal fasciculus R | 12,8645 |
|  | Uncinate fasciculus R | 2,3161 |
|  | Inferior fronto-occipital fasciculus R | 1,2903 |
|  | Superior longitudinal fasciculus (temporal part) R | 0,7742 |
|  | Cingulum (hippocampus) R | 0,7226 |
|  | Superior longitudinal fasciculus R | 0,6968 |
|  |  |  |
| Cluster 2 | Superior longitudinal fasciculus R | 10,0784 |
|  | Superior longitudinal fasciculus (temporal part) R | 3,0196 |
|  |  |  |
| Cluster 3 | Superior longitudinal fasciculus R | 0,8222 |
|  | Superior longitudinal fasciculus (temporal part) R | 0,0444 |

**Supplementary Table S4: Mean difference in FA between group (N= 25 participants with ASD and 24 healthy participants )**

^a^ Mean probabilities of the considered tract within the mask of results (outputs from ‘autoaq’ of FSL) in order of importance

|  | White-matter tracts  JHU white matter tractography atlas | Mean probabilities^a^ |
| --- | --- | --- |
| Cluster 1 | Forceps minor | 6,9 |
|  | Superior longitudinal fasciculus R | 5,8 |
|  | Inferior fronto-occipital fasciculus R | 2,3 |
|  | Superior longitudinal fasciculus (temporal part) R | 2,2 |
|  | Forceps major | 1,6 |
|  | Inferior longitudinal fasciculus R | 1,2 |
|  | Anterior thalamic radiation R | 0,8 |
|  | Cingulum (cingulate gyrus) L | 0,4 |
|  | Cingulum (cingulate gyrus) R | 0,1 |
|  | Uncinate fasciculus R | 0,1 |
|  |  |  |
| Cluster 2 | Inferior fronto-occipital fasciculus R | 13,7 |
|  | Inferior longitudinal fasciculus R | 9,8 |
|  | Superior longitudinal fasciculus R | 0,1 |
|  | Superior longitudinal fasciculus (temporal part) R | 0,1 |
|  | Uncinate fasciculus R | 0,1 |
|  |  |  |

**Supplementary figure 1**. Violin plot of the age distribution in each group


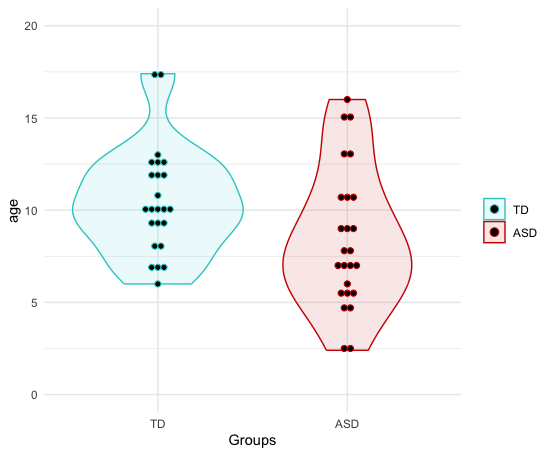


There was a significant age difference between groups (p = 0.04). This difference has been mostly accounted for by including age in our statistical models. Post-hoc analysis was performed dropping the two youngest subjects in the ASD group, making age difference no longer significant (p = 0.12). Results did not differ from the ones reported in the manuscript. Indeed, the correlation analyses between FA values and number of fixations to the eyes in the ASD group remained highly significant (b = 1.0e-03, t _(19)_ = 8.2; p = 1.1e-07), as remained the interaction (F _(1,41)_ = 52.5; p = 7.40e-09).

**Supplementary results regarding radial diffusivity (RD)**

RD values were obtained from clusters where a positive correlation between FA values and number of fixations to the eyes was observed.

**Supplementary Figure 2: Correlation between the number of fixations to the eyes and RD values in typically developing children, in children with ASD and interaction analysis (group by number of fixations to the eyes).**

**
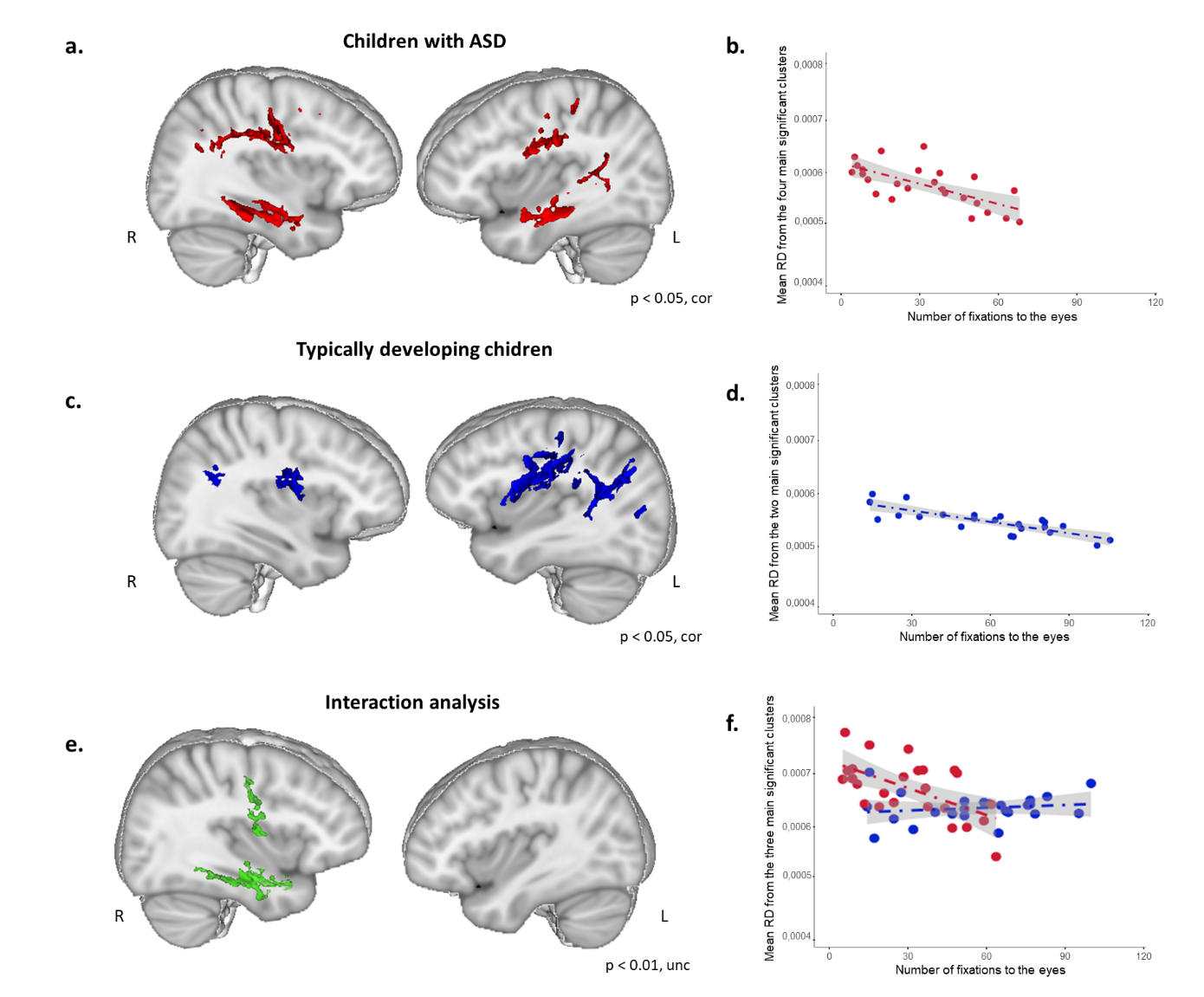
**

**(a) & (b)** *Participants with ASD.* **(a)** Significant negative correlation between the number of fixations to the eyes and RD values within the four main clusters where a positive correlation with FA values was described, namely in white matter tracts mainly encompassing right and left superior longitudinal fasciculus and right and left inferior longitudinal fasciculus. Results were overlaid on the MNI-152 template average brain using Mango software. Right and left sagittal views (x = 35, x = -38 respectively). **(b)** Scatterplot of negative correlation between the average RD from these clusters and the number of fixations to the eyes (b = -1.012, t(21) = -4.696; p = 0.000123, regression performed in R with age and sex as covariates).

**(c) & (d)** *Participants with typical development*. **(c)** Significant negative correlation between the number of fixations to the eyes and RD values within the two main clusters where a positive correlation with FA values was described, namely in white matter tracts mainly encompassing right and left superior longitudinal fasciculus. Results were overlaid on the MNI-152 template average brain using Mango software. Right and left sagittal views (x = 35, x = -38 respectively). **(d)** Scatterplot of negative correlation between the average RD from these clusters and the number of fixations to the eyes (b = -7.95e-07, t _(20)_ = -6.52; p = 2.36e-06, regression performed in R with age and sex as covariates).

**(e) & (f)** Interaction analysis. **(e)** Significant group by number of fixations to the eyes interaction within the three main clusters where an interaction was described regarding FA values, localized in white matter tracts mainly encompassing the anterior temporal portion of the right inferior longitudinal fasciculus. Results were overlaid on the MNI-152 template average brain using Mango software. Right and left sagittal views (x = 35, x = -38 respectively). **(f)** Scatterplot of significant group by number of fixations to the eyes interaction from these clusters (F_(1,43)_ = 14.5, p = 4.35 e-04, regression adding age and sex as covariates) performed in R.

**Supplementary results regarding mean diffusivity (MD)**

In typically developing participants, a significant negative correlation was observed between the number of fixations to the eyes and MD values within the two main clusters where a positive correlation with FA values was described, namely in white matter tracts mainly encompassing right and left superior longitudinal fasciculus (b = -3.22, t(20) = -3.255, p = 0.004).

No significant correlation was observed for participants with ASD and there was no group by number of fixations interaction regarding MD values.

**Supplementary Table S5: Effect of number of fixations to the eyes on FA values in the ASD group**

|  | **Estimate** | **Std. Error** | **t value** | **Pr(>\|t\|)** |  |
| --- | --- | --- | --- | --- | --- |
| Intercept | 4.372e-01 | 7.887e-03 | 55.430 | < 2e-16 | *** |
| Number of fixations to the eyes | 9.916e-04 | 1.357e-04 | 7.308 | 3.40e-07 | *** |
| Age | 5.223e-03 | 7.322e-04 | 7.133 | 4.92e-07 | *** |
| Sex | -2.575e-05 | 5.600e-03 | -0.005 | 0.996 |  |

**Supplementary Table S6: Effect of number of fixations to the eyes on FA values in the typically developing group**

|  | **Estimate** | **Std. Error** | **t value** | **Pr(>\|t\|)** |  |
| --- | --- | --- | --- | --- | --- |
| Intercept | 4.372e-01 | 1.158e-02 | 37.753 | < 2e-16 | *** |
| Number of fixations to the eyes | 8.806e-04 | 1.052e-04 | 8.369 | 5.77e-08 | *** |
| Age | 2.152e-03 | 9.545e-04 | 2.254 | 0.0355 | * |
| Sex | 7.324e-03 | 5.479e-03 | 1.337 | 0.1963 |  |

**Supplementary Table S7: Interaction between number of fixations to the eyes and group on FA values**

|  | **Sum Sq** | **df** | **F value** | **Pr (>F)** |  |
| --- | --- | --- | --- | --- | --- |
| Intercept | 0.309344 | 1 | 1806.8717 | <2.2e-16 | *** |
| Number of fixations to the eyes | 0.001381 | 1 | 8.0658 | 0.006864 | ** |
| Group | 0.006369 | 1 | 37.2025 | 2.621e-07 | *** |
| Age | 0.005675 | 1 | 33.1456 | 8.237e-07 | *** |
| Sex | 0.000139 | 1 | 0.8112 | 0.372790 |  |
| Number of fixations to the eyes by group | 0.008713 | 1 | 50.8932 | 8.237e-09 | *** |
| Residuals | 0.007362 | 43 |  |  |  |
